# Genetic Variants Associated with Life Expectancy in Patients with Chagas Disease

**DOI:** 10.3390/medsci14010137

**Published:** 2026-03-16

**Authors:** Mario Bruno Principato, Maria Victoria Carvelli, Analia Paolucci, Camila Principato, Rocio Villa Fernandez, Nicolas Aguirre, Gabriel Ercoli, Guillermo Alberto Keller, Guillermo Di Girolamo, Manuel Lago, Justo Carbajales

**Affiliations:** 1 Cardiology Department, Ramos Mejía Hospital, Ciudad Autónoma de Buenos Aires C1221ADC, Argentina; mbprincipato@yahoo.com.ar (M.B.P.);; 2 Argentine Society of Cardiology, Ciudad Autónoma de Buenos Aires C1115AAD, Argentina; 3 Cardiology Department, Facultad de Medicina, Universidad del Salvador, Ciudad Autónoma de Buenos Aires C1023AAB, Argentina; 4 Gempre, Genomics and Precision Preventive Medicine, Ciudad Autónoma de Buenos Aires C1087ACQ, Argentina; 5 Biotech Genetic Solutions—Oncological Genetics, Ciudad Autónoma de Buenos Aires C1048AAJ, Argentina; 6 Preventive Genetics Department, Austral University Hospital, Pilar B1629AHJ, Argentina; 7 Universidad de Buenos Aires, Facultad de Medicina, Ciudad de Buenos Aires C1121ABG, Argentina; gkeller@fmed.uba.ar; 8 Instituto Alberto C. Taquini de Investigaciones en Medicina Traslacional, Universidad de Buenos Aires, Ciudad Autónoma de Buenos Aires C1122AAJ, Argentina

**Keywords:** chagas, cardiomyopathy, survival, polymorphism, prognostic score

## Abstract

Single nucleotide polymorphisms (SNPs), as common genetic variations, can influence biological processes. Identifying these variations is crucial for recognizing high-risk subgroups, guiding preventive strategies, and enabling personalized management. **Objective:** This study aimed to determine the relationship between SNPs and survival, thereby identifying genetic profiles associated with increased risk. **Methods:** We included seropositive patients with Chagas disease who had a disease duration of >20 years and no comorbidities. DNA was extracted. A SNP panel focusing on genes involved in cardiac structure was created from the GnomAD database. Patients were followed for 8 years to assess survival. The association between SNPs and survival was evaluated, and a genetic risk score was generated. Univariate and multivariate Cox regression models assessed the association between SNPs (coded as ordinal variables) and survival time. SNPs with *p* < 0.05 were selected to construct a risk score, which was then assessed using Kaplan–Meier curves and median survival times. **Results**: A total of 182 patients were included, with 96.7% completing follow-up for a median of 5.1 years (interquartile range: 3.4–6.5). The median age was 62 years; 39.6% of patients were male, and 31% had reduced left ventricular ejection fraction. Univariate analysis showed that 3 of the 68 SNPs studied were associated with survival. Variant rs3755863 (*PPARGC1A* gene) was significantly associated with an increased risk of death (hazard ratio, HR = 1.94; *p* = 0.022). Conversely, two variants, rs7310615 (*SH2B3* gene) and rs7405731 (*JUP* gene), showed a protective effect with significantly reduced mortality risk (HR = 0.45; *p* = 0.006 and HR = 0.48; *p* = 0.006, respectively). In multivariate analysis, rs7310615 and rs7405731 remained significantly associated with survival. A genetic risk score was constructed, assigning 0 points for homozygous wild-type, 1 point for heterozygotes, and 2 points for homozygous alternative alleles. Individual scores were calculated, and survival was estimated for each score category using Kaplan–Meier analysis and median survival times. **Conclusions:** Two SNPs were identified as significantly associated with survival. These findings require confirmation in larger and more diverse populations. Their validation could enable the identification of a subgroup of patients at particularly high risk.

## 1. Introduction

Since 2005, the World Health Organization (WHO) has included Chagas disease on its list of Neglected Tropical Diseases. It is estimated that more than seven million people worldwide are infected with *T. cruzi*, resulting in approximately 10,000 deaths annually. Despite its increasing global prevalence, Chagas disease is primarily found in endemic areas of 21 countries in Latin America [1], where its transmission is largely linked to the presence of the insect vector. Currently, it is estimated that more than 100 million people are at risk of infection. The insect vector is hematophagous and, when infected with the parasite, transmits the infection through contaminated feces. The parasite can enter the human body through wounds in the skin or mucous membranes (vector-borne transmission). Other routes of transmission include blood transfusions, organ transplants, vertical transmission during pregnancy or childbirth, and consumption of food or beverages contaminated with fecal matter from infected insects [2].

Chagas disease is responsible for the non-ischemic dilated cardiomyopathy with the highest morbidity and mortality in Latin America. It has two clinical phases: an acute phase, which is usually asymptomatic in 95% of patients, and a chronic phase with a prolonged latency period of 10–30 years. In this latter group, between 25% and 30% of individuals develop signs or symptoms of cardiac involvement (Chagasic cardiomyopathy) [3].

In recent decades, Chagas disease has expanded globally due to migratory movements, becoming an emerging worldwide health issue. Despite a shared etiological agent, the clinical progression of infected patients is strikingly diverse [4]. While some individuals remain asymptomatic throughout their lives, others develop serious cardiac complications, such as heart failure, malignant arrhythmias, or sudden death [5]. This diversity suggests that, in addition to environmental, immunological, and parasite-based factors, host genetic determinants play a role in disease progression.

However, there is no consensus on risk indicators for the development of this condition and other cardiovascular events, independent of the effect of traditional risk factors [6]. In this context, single nucleotide polymorphisms (SNPs) have been extensively researched as markers of susceptibility, clinical progression, and therapeutic response in various complex diseases, including cardiovascular disorders [7]. SNPs are the most common form of genetic variation in the human population and can influence the expression or function of genes involved in fundamental processes such as inflammation, cardiac remodeling, fibrosis, and cellular apoptosis [8].

While some studies have explored the association between specific SNPs and the development of cardiac manifestations in Chagas disease, the impact of these genetic variants on long-term survival remains to be fully elucidated. Identifying SNPs associated with survival could provide valuable information, leading to a more comprehensive understanding of the biological mechanisms that influence patient prognosis.

The aim of this study was to identify genetic variants (not related to familial pathologies) associated with survival in patients with positive serology for Chagas disease.

## 2. Methods

This single-center study included subjects aged 21 to 80 years from provinces in Northwest Argentina and Southern Bolivia who tested positive for Chagas disease. All participants had a documented chronic *Trypanosoma cruzi* infection exceeding 20 years, confirmed by serological tests and treating physicians. They had also resided outside endemic regions for at least two decades prior to enrollment.

Exclusion criteria included failure to provide informed consent; presence of other serious non-cardiovascular diseases or a life expectancy of less than one year; participation in other research protocols within the last 30 days; lack of reliable personal or telephone contact; history of alcohol or drug abuse within the last 6 months; evidence of liver failure (transaminases > 3× upper limit of normal or bilirubin > 2 mg/dL); treatment with drugs known to have cardiovascular effects (immunosuppressants, nitrates, estrogens); diagnosis of acute or chronic coronary artery disease; or indication for revascularization, percutaneous coronary intervention, or coronary artery bypass surgery in the previous 6 months. Subjects with renal failure (serum creatinine levels > 2.5 mg/dL), severe obstructive pulmonary disease, dilated cardiomyopathy of other etiologies (e.g., rheumatoid arthritis, diabetes mellitus, hypertension), significant valvular heart disease (except those secondary to mitral and/or tricuspid annular dilation), ventricular pacing > 50%, or autoimmune diseases (e.g., systemic lupus erythematosus, scleroderma, or hepatitis C), esophageal or digestive disorders were also excluded. The inclusion and exclusion criteria were evaluated in all patients. Patients who met all the criteria were enrolled in the study.

Genomic DNA extraction and initial processing were conducted at a specialized national center to ensure the preservation of backup samples. Subsequently, specimens were sent via certified courier, in accordance with international regulations, to the Xenética Cardiovascular laboratory (Instituto de Investigación Sanitaria de Santiago, Complexo Hospitalario Universitario de Santiago de Compostela) for genotyping and analysis. The genotyping team was blinded to the participants’ clinical characteristics to ensure masking of information for statistical analysis.

A polygenic panel was selected comprising genes related to ventricular contraction and relaxation (*TTN*, *BAG3*, *MTSS1*) [9,10,11,12,13,14,15,16], myocardial metabolism (*PPARGC1A*, *SIRT1*, *AKT1 mTOR*, *AMPK*, *PRKAA2*) [17], adrenoceptor beta 1 (*ADRB1*) [18,19,20,21,22], cholinergic receptor muscarinic 2 (*CHRM2*), angiotensin II receptor type 1 (*AGTR1B*) [23,24], natriuretic peptide A (*NPPA*), cell cycle regulators (*CDKN1A*, *RYR2*), sarcomeric structure (*ATP2A2*, *DSP*, *JUP*) [25,26,27], cellular energy (*PRKAB2*, *PRKAB1*) [28], mitochondrial activity (*SOD 2*), apoptosis (*AKT1*), regulator of cellular adaptive responses to hypoxia (*HIF1A*), respiratory chain (*LDHA*), nitric oxide signaling (*NOS1*, *NOS2P3*), and Growth factors and inflammation signaling (*SH2B3*).

SNP selection was based on the gnomAD resource. We prioritized common variants by allele frequency (target range approximately 10–40% according to gnomAD population frequencies) and evaluated quality metrics reported in gnomAD v4, retaining variants with a PASS filter status. As shown in the extracted gnomAD v4 checklist (Supplementary Table S1 SNPs analyzed), the vast majority of variants displayed a PASS filter status in both exomes and genomes. For multiallelic sites, gnomAD reports multiple alternative alleles as distinct variant entries; in these cases, we focused on the alternative allele corresponding to the selected rsID and target frequency range, while additional alternate alleles at the same genomic position were rare or absent in the corresponding dataset (e.g., very low allele frequency or allele count equal to zero) and therefore not relevant for panel design.

After genotyping, Hardy–Weinberg equilibrium (HWE) was formally evaluated in our study population. Genotyping was performed using iPLEX Gold technology, and deviation from HWE was tested using the SNPassoc package in R across the full cohort, as a post-genotyping quality control step prior to association analyses. All analyzed SNPs met Hardy–Weinberg equilibrium criteria and no variants were excluded on the basis of HWE deviation. Finally, 68 SNPs were analyzed.

The association between SNPs, coded as an ordinal variable (0 for homozygous wild-type, 1 for heterozygous, and 2 for homozygous alternative alleles), was evaluated.

First, each SNP underwent univariate analysis using the Cox proportional hazards model to assess its predictive value for survival time. Survival time was calculated as the difference between the date of last patient contact and their date of birth. SNPs with a *p*-value < 0.05 were considered significantly associated with survival and were subsequently included in a multivariate Cox regression model. Results were expressed as hazard ratios (HR) and their 95% confidence intervals (CI). Our objective was to identify single nucleotide polymorphism genetic variants that predict mortality, so it was not adjusted for other post-exposure factors, since on the one hand, if they were dependent on the variant and were associated with mortality, they would be causal mediators and would produce bias in the estimation of the association (z1) and, if they were not related to the exposure, they would not modify the estimate but would increase the variance, reducing the power of the study (z2, z3). Second, a risk score was developed using SNPs significantly associated with survival time for the prediction model. Finally, the score was calculated for each analyzed subject. Survival was estimated using Kaplan–Meier curves, and median survival time was calculated for each risk score category.

All analyses were performed using R^®^ 4.1.1 software (R Development Core Team/R Foundation for Statistical Computing, Vienna, Austria).

The study was conducted in accordance with the Declaration of Helsinki, and approved by the institutional ethics committee of the Hospital General de Agudos Ramos Mejía, Buenos Aires, Argentina; approval code AP2016050401, date of approval 4 May 2016. Signed informed consent was obtained from all subjects involved in the study.

## 3. Results

A total of 182 subjects with positive serology for Chagas disease were analyzed. Baseline characteristics of the study population are described in Table 1.

Patient inclusion was initiated in January 2016 and completed in 2020. The follow-up was planned for 8 years (for this analysis, a survival cutoff was performed in January 2025) for a median of 5.1 years (interquartile range [IQR]: 3.4–6.5 years) after their first visit.

**Figure 1:**
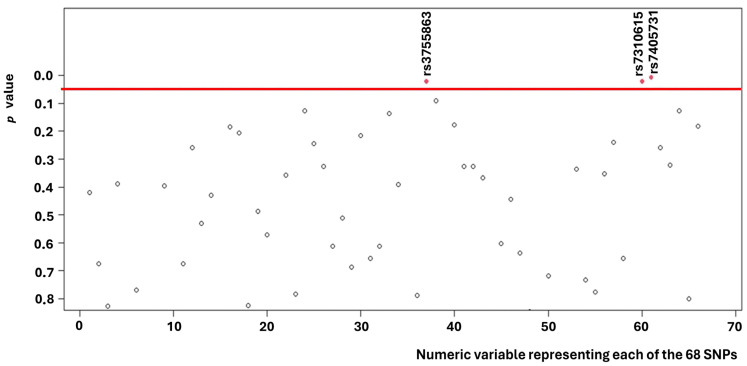
*p*-values found for each of the 68 SNPs.

Univariate analysis revealed that three of the 68 SNPs (Figure 1) analyzed were associated with survival (Supplementary Table S1).

**Figure 2:**
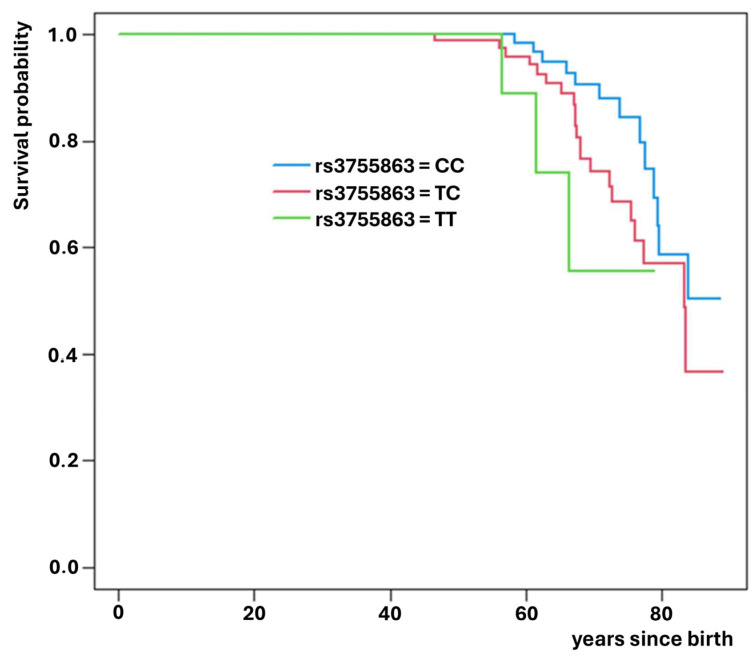
Survival curves for SNP rs3755863 which reduces expected survival.

**Figure 3:**
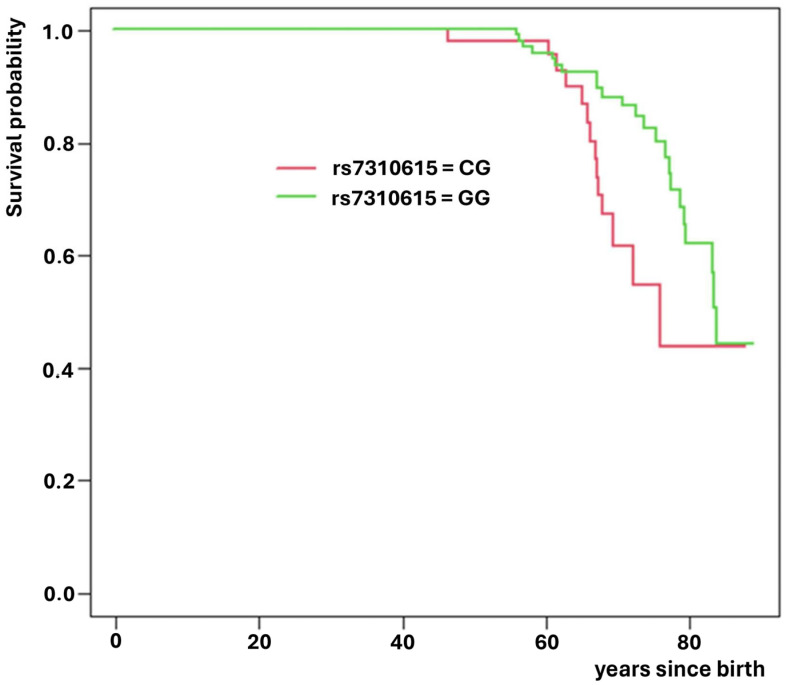
Survival curves for SNP rs7310615 which increases expected survival.

**Figure 4:**
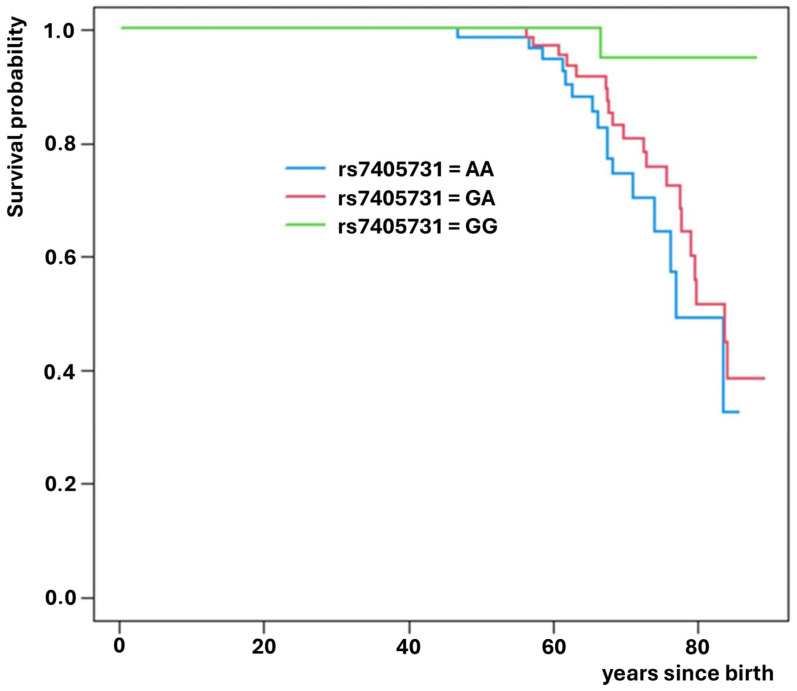
Survival curves for SNP rs7405731 which increases expected survival.

Of these, rs3755863 (*PPARGC1A* gene) significantly increased the risk of death (HR = 1.94; *p* = 0.022, Figure 2), while rs7310615 (*SH2B3* gene) and rs7405731 (*JUP* gene) exhibited protective effects, reducing the risk of death (HR = 0.45; *p* = 0.006; Figure 3 and HR = 0.48; *p* = 0.006; Figure 4, respectively).

Multivariate analysis including the three SNPs showed that only rs7310615 and rs7405731 remained significantly associated with survival, each exhibiting a substantial reduction in the risk of death (exceeding 50% per alternative allele). The corresponding HRs are presented in Table 2.

**Figure 5:**
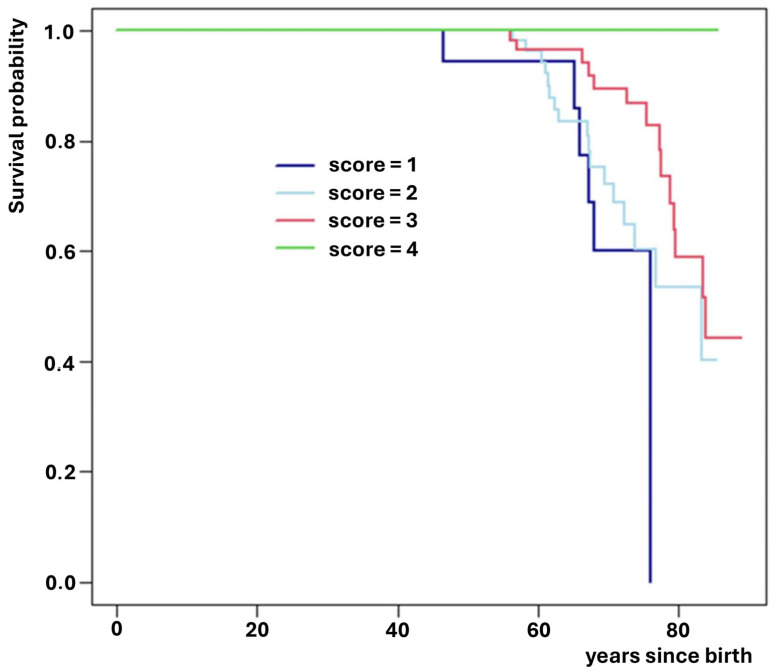
Survival for each score category.

As previously described, a genetic risk score was developed using the significantly associated SNPs, with points assigned as follows: 0 for homozygous wild-type, 1 for heterozygous, and 2 for homozygous alternative alleles (Table 3). The principle used in assigning the score is based on the theoretical support that treats single nucleotide polymorphism variants as an ordinal variable, supported by the Hazard ratio obtained, which indicates a proportional and linear increase in risk for each variant category. The score was calculated for each subject, and survival was estimated using Kaplan–Meier curves (Figure 5). Median survival time was then calculated for each risk score category (Table 4).

### Bioinformatics Analysis

The two variants were characterized using dbSNP, CADD, PhyloP, SpliceAI, and VEP. Neither variant was predicted to affect splicing or protein structure. This suggests they may be genetic markers in linkage disequilibrium with as-yet-unidentified functional variants.

## 4. Discussion

The heterogeneity in clinical course and survival among patients with cardiac disease, such as Chagas-associated dilated cardiomyopathy, reflects the complex interplay of genetic and environmental factors that modulate individual responses to myocardial damage. In this context, genetic polymorphisms, particularly SNPs, are potential markers for identifying subgroups with different prognoses and for understanding underlying pathogenic mechanisms.

In our study, two genetic variants demonstrated an independent association with survival: rs7310615 (located in the *SH2B3* gene) and rs7405731 (in the *JUP* gene). Both exhibited a protective effect, being significantly associated with a reduced risk of death. This suggests a potential relationship that could contribute to mechanisms modulating the progression of heart failure or resistance to chronic myocardial damage.

The *SH2B3* gene encodes the SH2B3 protein (LNK), which acts as a negative regulator in several cell signaling pathways, including those triggered by cytokine receptors [29]. Its role in immune and hematopoietic regulation is well documented, participating in inflammatory response modulation [30]. Given that chronic inflammation is a key factor in ventricular remodeling and the progression of heart failure, variants in *SH2B3* have the potential to influence the inflammatory response and, consequently, the clinical course.

In addition, *SH2B3* regulates intracellular pathways related to JAK/STAT and MAPK/ERK, which are directly involved in cell survival, proliferation, and apoptosis [31]. The intronic or synonymous rs7310615 variant could affect regulatory regions of the gene or be in linkage disequilibrium with other functional variants that modulate *SH2B3* expression or function [32]. Research in animal models has demonstrated that *SH2B3* alterations impact the cardiovascular stress response, thereby supporting its role in heart failure modulation [33].

The *JUP* gene encodes plakoglobin, an essential component of desmosomes and adherens junctions in cardiomyocytes. These structures are critical for myocardial mechanical and electrical integrity. The stability and intercellular communication maintained by plakoglobin ensure synchronized cardiac contraction and resistance to mechanical stress [34]. While the rs7405731 variant does not alter the protein sequence, it has the potential to affect *JUP* gene regulatory elements, thereby influencing its expression [32,35].

Alterations in *JUP* and other desmosomal genes have been implicated in arrhythmogenic and dilated cardiomyopathies, impacting the predisposition to arrhythmia and heart failure [34]. Consequently, protective variants, such as rs7405731, could potentially enhance myocardial structural preservation, thereby contributing to longer survival.

Both variants underwent thorough bioinformatics analysis. The rs7405731 variant was classified as synonymous, with no expected changes in protein structure. Nevertheless, its potential effects on RNA splicing and evolutionary conservation were explored. SpliceAI, CADD, and PhyloP did not predict significant functional alterations [36,37,38]. A conservation analysis using a LOGO sequence plot, generated from data of 100 vertebrate species, showed partial conservation of the adenine base (A) at that position, with a preponderance of guanine (G), but without strong conservation of any base suggestive of a deleterious effect.

The rs7310615 variant (NM_005475.3:c.732+8368C>G) is located in a deep intronic region of *SH2B3*, with no direct impact on amino acid coding. SpliceAI, CADD, and PhyloP models were unable to predict any relevant deleterious effect on splicing or protein structure [36,37,38]. Analysis revealed a conspicuous absence of marked conservation of the cytosine base (C), accompanied by a notable presence of guanine (G), though not at levels considered pathogenic.

Consequently, bioinformatics analysis suggests that these variants do not induce overt direct functional protein alterations or splicing. This finding suggests their association with survival is likely attributable to their role as markers in linkage disequilibrium with as-yet-unidentified functional variants [39,40,41,42]. Therefore, complementing these findings with additional functional and genomic tests is essential to clarify the molecular mechanisms involved.

The objective of the present study was to construct a predictive survival model based on one or more SNPs. A multivariable Cox proportional hazards regression model without Bonferroni correction was used. This decision was guided by three key considerations: (1) prioritizing sensitivity over specificity in the identification of predictors, as the small sample size would have substantially reduced statistical power and potentially precluded the identification of any significant associations; (2) the targeted evaluation of 68 SNPs selected based on biological plausibility rather than random screening; and (3) the aim of estimating the magnitude of the association between each SNP and the study outcome. A total of 68 SNPs were evaluated, thereby increasing the risk of type α error. Given the limited sample size, only model derivation was performed. Therefore, the findings should be externally validated in an independent cohort to assess the score’s performance. Accordingly, due to sample size limitations, this study should be considered exploratory, and further validation in a separate population is required to confirm the role of these genetic variants. Given the observational nature of the study, it is imperative to acknowledge potential biases inherent in such research methodologies. First, immortal time bias must be considered. Patients included in the study had to survive long enough to become infected and seek medical care when their follow-up began. Consequently, patients were subject to immortal time bias during the period between birth and study entry, meaning SNPs associated with high mortality in the first years of life could not have been identified.

## 5. Study Limitations

Given the observational nature of the study, it is imperative to acknowledge potential biases inherent in such research methodologies.

First, the primary limitation of this study is its sample size. While sufficient for detecting significant associations with survival, it limits the power to detect gene–gene or gene–environment interactions. Second, given the focus on a relatively geographically and genetically homogeneous cohort, additional validation studies are necessary to generalize the findings to other populations. Third, direct functional tests to validate the biological effect of the observed variants were not performed. Therefore, the results should be interpreted as statistical associations requiring experimental confirmation. Finally, other possible structural or non-coding variants that could influence the same genes or metabolic pathways were not analyzed. The evaluation of 68 SNPs introduced an elevated risk of alpha error. Due to the limited number of participants, we exclusively conducted the prediction model’s derivation. Consequently, these results require validation in an independent cohort to assess the score’s performance and confirm the variants’ role.

With 68 variables tested, both Bonferroni and Benjamini–Hochberg false discovery rate adjustments would require a significance threshold of approximately *p* < 0.0007 (0.05/70). To detect a hazard ratio of 0.5 (i.e., a 50% reduction in mortality—an effect size considered highly suggestive of causality under GRADE criteria), a total of 274 events would be required to achieve adequate statistical power. Given the event rate observed in our cohort, this would necessitate enrolling more than 1300 patients during the study period. Therefore, applying stringent multiple-testing corrections would have rendered the detection of clinically meaningful associations virtually impossible. Accordingly, this study should be considered exploratory and hypothesis-generating, and its findings require confirmation in larger, independent cohorts. For these reasons, Bonferroni correction was not applied. The multivariate Cox regression model without Bonferroni adjustment was employed. This decision was guided by three key considerations: (1) the prioritization of sensitivity over specificity in the identification of predictors, (2) the specific exploration of 68 SNPs rather than a random selection, driven by the need to evaluate their biological support, and (3) the estimation of the magnitude of the association of each SNP with the outcome under study.

For confounding to occur, a variable must be causally associated with both the exposure and the outcome, and it must precede both. In the context of germline genetic variants, no clinical variable fulfills this criterion, as SNPs are determined at conception and therefore precede the development of any postnatal clinical condition. Accordingly, no observable clinical variable can act as a traditional confounder of the association between the SNPs and mortality.

Therefore, no adjustment was made for post-exposure clinical factors. If such factors were influenced by the genetic variant and associated with mortality, they would represent causal mediators, and adjusting for them would introduce bias into the association estimate [43]. Conversely, if these factors were not associated with the exposure, adjustment would not materially change the effect estimate but would increase model variance and reduce statistical power [44,45].

## 6. Conclusions

Our study identified significant associations between SNPs rs7310615 (*SH2B3*) and rs7405731 (*JUP*) and increased survival in patients with Chagas disease. Bioinformatics analysis suggests these variants do not directly modify protein structure or splicing. Instead, they likely act as markers in linkage disequilibrium with other as-yet-unidentified functional variants. These results offer a promising avenue for future research into the molecular mechanisms explaining the clinical heterogeneity and survival in Chagas disease, with potential implications for risk stratification and the development of personalized therapies.

## Figures and Tables

**Table 1:** Baseline characteristics of patients.

Age (mean ± SD years)		61.1 ± 11.2
Gender—Male		72 (39.6%)
Reduced Left Ventricular Ejection Fraction		53 (30%)
Hypertension		48 (27%)
Diabetes	No	1617 (92%)
	Type 1	3 (2%)
	Type 2	11 (6%)
Heart Failure		38 (21%)
Heart blocks		95 (53%)

The data show the number of subjects who have the condition, and in parentheses the percentage they represent of the studied population, except for age, where the average and standard deviation are shown.

**Table 2:** Hazard Ratio associated with SNPs.

SNP	Hazard Ratio	95% CI
rs7310615	0.396	0.196–0.798
rs7405731	0.466	0.278–0.779

**Table 3:** Risk score.

rs7310615 = CC rs7310615 = CG rs7310615 = GG	0 points 1 point 2 points
rs7405731 = AA rs7405731 = GA rs7405731 = GG	0 points 1 point 2 points

**Table 4:** Median Survival associated with score.

Score	Median Survival (Years)
1	75.98
2	83.41
3	83.83
4(*)	>85.51

(*) Since no subjects in category 4 died by the end of the follow-up period, the median survival cannot be estimated and is censored at a value greater than 85.5 years.

## Data Availability

The original contributions presented in this study are included in the article/Supplementary Materials. Further inquiries can be directed to the corresponding author.

## Supplementary Materials

The following supporting information can be downloaded at: https://www.mdpi.com/article/10.3390/medsci14010137/s1, Supplementary Table S1: Complete list of SNPs analyzed, and Supplementary Table S2: Complete dataset with the raw data from this study. The identity of the participating patients is coded to protect their privacy.
