# Supplementary material for: Genetic Variants Associated with Life Expectancy in Patients with Chagas Disease
Source: Med Sci (Basel). 2026 Mar 16;14(1):137. doi: 10.3390/medsci14010137 (PMC13027560; doi:10.3390/medsci14010137)
Supplement: Supplementary file 1 [file medsci-14-00137-s001.zip › Table S1 Supplementary annex.pdf]

Supplementary annex S1: SNPs analyzed.

GENE	SNP	
ADRB1	rs12414657	upstream variant
	rs1801252	missense variant
	rs1801253	missense variant
	rs3813719	Downstream Variant
	rs3813720	Downstream Variant
CHRM2	rs13247260	upstream intron
	rs6962027	Prime UTR Variant
	rs6967953	Prime UTR Variant
NOS1	rs12811583	intron
	rs1875140	intron
	rs3741475	Synonymous Variant
TTN	rs2042995	missense variant
	rs2255167	intron
PRKAA2	rs17848595	synonymous variant
	rs61772962	Intron
	rs17848596	missense variant
MTOR	rs1034528	Intron
	rs11581010	Intron
	rs17036350	Intron
	rs74225573	Intron
RYR2	rs10802607	Intron
	rs67622164	Intron
	rs10925391	Intron
	rs16835818	Intron
JUP	rs1126821	Missense
	rs8067890	Intron
	rs7405731	missense variant
	rs7216034	Intron
SOD2	rs11752345	Intron
	rs4880	missense variant
PRKAB2	rs1348316	Intron
	rs72708505	Intron
CDKN1A	rs146170154	Intron
ZNF592	rs149369954	noncoding transcript variant
BAG3	rs1831018	BAG cochaperone 3
	rs72840788	Intron
	rs7071853	
ATP2A2	rs1860561	Intron
DSP	rs2076300	synonymous variant
	rs926411	Intron
SIRT1	rs2236318	Intron
	rs2273773	synonymous variant
PPARGC1A	rs2290604	Intron
	rs3755863	synonymous variant
AGTR1	rs275653	Upstream variant
	rs5186	3 Prime UTR Variant
	rs387967	Upstream variant

	rs422858	Upstream variant
SOD2	rs2758332	Intron
	rs5746094	Intron
PRKAB1	rs278145	intron
	rs278149	intron
PPARGC1A	rs2946385	Stop gained
	rs8192678	Missense Variant
PRKAB2	rs34838459	Synonymous variant
LOC105375743	rs34866937	intron
AKT1	rs3730346	intron
	rs3730358	intron
	rs3803304	intron
HIF1A	rs373909145	intron
NPPA	rs5063	Missense Variant
	rs5064	Intron
	rs5065	stop loss
DERL32KB	rs6003909	Upstream Variant
NOS2P3	rs62066941	Intron
LDHA	rs6498	Synonymous Variant
SH2B3	rs7310615	Intron
SIRT1	rs7896005	Intron
CHRM2	rs8191992	3 Prime UTR Variant
CLCNKA2KB	rs945425	Upstream Variant
